# Resident Physician Recognition of Tachypnea in Clinical Simulation Videos in Japan: Cross-Sectional Study

**DOI:** 10.2196/72640

**Published:** 2025-07-31

**Authors:** Kiyoshi Shikino, Yuji Nishizaki, Sho Fukui, Koshi Kataoka, Daiki Yokokawa, Taro Shimizu, Yu Yamamoto, Kazuya Nagasaki, Hiroyuki Kobayashi, Yasuharu Tokuda

**Affiliations:** 1 Department of Community-Oriented Medical Education, Graduate School of Medicine, Chiba University, 1-8-1, Inohana, Chu-ou-ku, Chiba, 2608670, Japan, 81 43-222-7171; 2 Department of General Medicine, Chiba University Hospital, Chiba, Japan; 3 Division of Medical Education, School of Medicine, Juntendo University, Tokyo, Japan; 4 General Medicine, School of Medicine, Kyorin University, Tokyo, Japan; 5 Department of Diagnostic and Generalist Medicine, Dokkyo Medical University, Mibu, Japan; 6 Division of General Medicine, Center for Community Medicine, Jichi Medical University, Shimotsuke, Japan; 7 University of Tsukuba, Tsukuba, Japan; 8 Department of Internal Medicine, Mito Kyodo General Hospital, Mito, Japan; 9 Muribushi Okinawa Center for Teaching Hospitals, Okinawa, Japan; 10 Tokyo Foundation for Policy Research, Tokyo, Japan

**Keywords:** clinical competence, clinical simulation video, general medicine in-training examination, non-verbal information, tachypnea

## Abstract

**Background:**

Traditional assessments of clinical competence using multiple-choice questions (MCQs) have limitations in the evaluation of real-world diagnostic abilities. As such, recognizing non-verbal cues, like tachypnea, is crucial for accurate diagnosis and effective patient care.

**Objective:**

This study aimed to evaluate how detecting such cues impacts the clinical competence of resident physicians by using a clinical simulation video integrated into the General Medicine In-Training Examination (GM-ITE).

**Methods:**

This multicenter cross-sectional study enrolled first- and second-year resident physicians who participated in the GM-ITE 2022. Participants watched a 5-minute clinical simulation video depicting a patient with acute pulmonary thromboembolism, and subsequently answered diagnostic questions. Propensity score matching was applied to create balanced groups of resident physicians who detected tachypnea (ie, the detection group) and those who did not (ie, the non-detection group). After matching, we compared the GM-ITE scores and the proportion of correct clinical simulation video answers between the two groups. Subgroup analyses assessed the consistency between results.

**Results:**

In total, 5105 resident physicians were included, from which 959 pairs were identified after the clinical simulation video. Covariates were well balanced between the detection and non-detection groups (standardized mean difference <0.1 for all variables). Post-matching, the detection group achieved significantly higher GM-ITE scores (mean [SD], 47.6 [8.4]) than the non-detection group (mean [SD], 45.7 [8.1]; mean difference, 1.9; 95% CI, 1.1‐2.6; *P*=.041). The proportion of correct clinical simulation video answers was also significantly higher in the detection group (39.2% vs 3.0%; mean difference, 36.2%; 95% CI, 32.8‐39.4). Subgroup analyses confirmed consistent results across sex, postgraduate years, and age groups.

**Conclusions:**

Overall, this study revealed that detecting non-verbal cues like tachypnea significantly affects clinical competence, as evidenced by higher GM-ITE scores among resident physicians. Integrating video-based simulations into traditional MCQ examinations enhances the assessment of diagnostic skills by providing a more comprehensive evaluation of clinical abilities. Thus, recognizing non-verbal cues is crucial for clinical competence. Video-based simulations offer a valuable addition to traditional knowledge assessments by improving the diagnostic skills and preparedness of clinicians.

## Introduction

Detecting non-verbal cues is an essential skill that resident physicians must acquire during their clinical training. However, accurately assessing detection ability is challenging [1]. In traditional text-based examinations, non-verbal information, such as respiratory rate or breathing patterns, is written, making true recognition of these cues clear.

The Japan Institute for Advancement of Medical Education Program (JAMEP) created the General Medicine In-Training Examination (GM-ITE) to evaluate resident physicians’ clinical knowledge across Japan [2]. This 2-hour exam comprising 80 multiple-choice questions (MCQs) [2] provides practical feedback on training programs by identifying areas for improvement through objective and reliable clinical competency assessment [3]. The GM-ITE is a validated examination that effectively assesses core medical knowledge and skills. However, its text-based design makes it difficult to evaluate the examinee’s ability to detect non-verbal cues [4]. A sample GM-ITE question illustrating the typical MCQ format and content is provided in Multimedia Appendix 1 [5].

To address this gap, we developed and integrated a clinical simulation video into the GM-ITE [6], offering an effective assessment tool by providing contextualized real-world scenarios that require resident physicians to apply knowledge dynamically, mirroring actual patient interactions [7,8]. By incorporating non-verbal information with verbal cues, video simulation offers a more authentic evaluation of clinical practice. This simulation involved a medical interview and a physical examination scenario, requiring participants to actively identify critical non-verbal cues, such as tachypnea. The clinical simulation video successfully assessed multiple domains of clinical competencies [8]. However, the association between the ability to detect non-verbal cues and clinical competency remains unknown.

Herein, we explored whether the ability to detect non-verbal cues, like tachypnea, in the clinical simulation video correlates with higher GM-ITE scores and correct clinical simulation video answers. We hypothesized that resident physicians who could accurately identify tachypnea would demonstrate superior clinical competence, yielding higher GM-ITE scores.

## Methods

### Study Design, Setting, and Participants

We conducted a multicenter cross-sectional study of GM-ITE for the academic year 2022 (GM-ITE 2022). GM-ITE employs a methodology similar to that of internal medicine (IM)-ITE. In Japan, the GM-ITE 2022 includes many residency programs in teaching hospitals, with other hospitals allowing voluntary participation. Consequently, 9011 resident physicians from 662 Japanese hospitals participated between January 17 and January 30, 2023. After the final question, participants were asked to voluntarily watch the clinical simulation video to provide a diagnosis, followed by a questionnaire to collect information on demographics and residency programs. This study included all resident physicians who participated in GM-ITE 2022 at one of the study sites, excluding those who did not participate in or answer the clinical simulation video question, or did not provide informed consent for study participation.

### Ethical Considerations

This study was approved by the Ethical Review Committee of the Japan Organization for Advancing Medical Education (approval no. 23‐26). All participants provided informed consent before participating. The study was conducted in accordance with the ethical standards and principles of the Declaration of Helsinki. Informed consent was obtained from all participants for the publication of identifying information in an online open-access publication. All participants read and signed an informed consent form before participating. To ensure confidentiality, all data were anonymized prior to analysis. No compensation was provided for involvement in the study. In accordance with ethical standards and journal policies, we obtained explicit informed consent from all actors appearing in the video material associated with this study. The actors acknowledged and agreed that the videos would be published as part of the study.

### Procedures

#### Innovative Examination Using High-Quality Video Simulation

This study used a clinical simulation video developed and introduced in a prior study [9]. In the 5-minute video filmed from a resident physician’s perspective, the resident physician conducted a medical interview and physical examination, with the camera capturing the patient’s and family members’ verbal and non-verbal responses. The video depicts a 40-year-old male patient with a recent history of lower limb fracture who presented to the emergency room with fainting as the chief concern. The correct diagnosis was acute pulmonary thromboembolism. This scenario was designed to challenge residents’ clinical reasoning skills. The patient exhibited physical signs, including tachypnea, jugular venous distension, and a right lower limb fracture, with actual heart sounds presented during auscultation. Crucially, tachypnea (28 breaths per minute) and respiratory patterns were not highlighted in the text, but presented as non-verbal cues, requiring participants to recognize these signs through observation, without explicit textual focus.

The video was produced by a professional television production company under the guidance of three JAMEP medical supervisors and three authors who ensured the medical accuracy and educational relevance. The use of professional actors and their added effects, such as heart sounds, enhanced the scenario realism of the scenario and aligned it with the objectives of clinical residency training in Japan.

After watching the clinical simulation video, participants answered the following clinical simulation video questions [10]:

Q1. Please state the most likely diagnosis for this patient (free text).

Q2. Please state the three pertinent positive clinical information related to the most likely diagnosis (free text).

For Q1, multiple disease name patterns indicating “acute pulmonary thromboembolism” (eg, acute pulmonary embolism or pulmonary thromboembolism) were accepted as correct. For Q2, the pertinent positive clinical findings included tachypnea, jugular venous distension, and a history of right lower limb fracture. Answers were evaluated based on whether key clinical signs were correctly identified, further contributing to diagnostic ability assessment. Two authors independently assessed, discussed, identified, and agreed upon the answers. The interrater reliability was measured using the κ coefficient, which indicated almost perfect agreement for Q1 (κ=0.91) and substantial agreement for Q2 (κ=0.82) [11].

#### Data Collection 

This study used information from the GM-ITE score and questionnaire, including sex, postgraduate year (PGY 1 or 2), rotation in the general medicine department, duration of rotation, number of monthly night shifts, average number of assigned inpatients, self-study time per day, and categorized weekly duty hours.

#### Exposure and Outcome Measurement Data

The exposure of interest was whether the resident physicians detected tachypnea in the innovative examination clinical simulation video. This non-verbal cue was critical for the correct diagnosis. The primary outcome was the total GM-ITE score, which ranged from 0 to 80 points. The secondary outcome included the results of the clinical simulation video (correct or incorrect).

### Statistical Analyses

Initially, we described participant-level information by groups that detected tachypnea (ie, tachypnea-detection group) and did not detect tachypnea (ie, tachypnea-non-detection group) in the clinical simulation video using appropriate summary statistics.

To adjust for potential confounders, we conducted propensity score matching analysis. Propensity scores for the probability of detecting tachypnea were calculated using a logistic regression model incorporating sex, PGY, rotation in the general medicine department, rotation duration, number of night shift duties per month, average number of assigned inpatients, self-study time, and weekly duty hours. We applied a greedy matching algorithm using a logit of propensity scores with calipers equal to 0.2 of the standard deviation of logit-propensity scores [12]. We assessed the balance of covariates between the groups before and after matching using the standardized mean difference (SMD) [13]. Covariates were considered well-balanced if the absolute SMD was <0.1.

The GM-ITE scores and clinical simulation video results were compared before and after propensity score matching. T-tests were applied to assess differences in the GM-ITE scores, while the *χ*^2^ test was used for the clinical simulation video results. Stratified subgroup analyses by gender (male or female), grade (PGY-1 or 2), and age (unknown, <27 years, and ≥27 years) were conducted to evaluate robustness.

All analyses were conducted using the Statistical Analysis System version 9.4 for Windows (SAS Institute Inc., Cary, NC, USA) and JMP version 17 for Windows (SAS Institute Inc., Cary, NC, USA) according to the Strengthening the Reporting of Observational Studies in Epidemiology guidelines. A complete case analysis was performed. The mean differences and 95% CI were reported for all analyses.

## Results

### Baseline Characteristics

**Figure 1.**
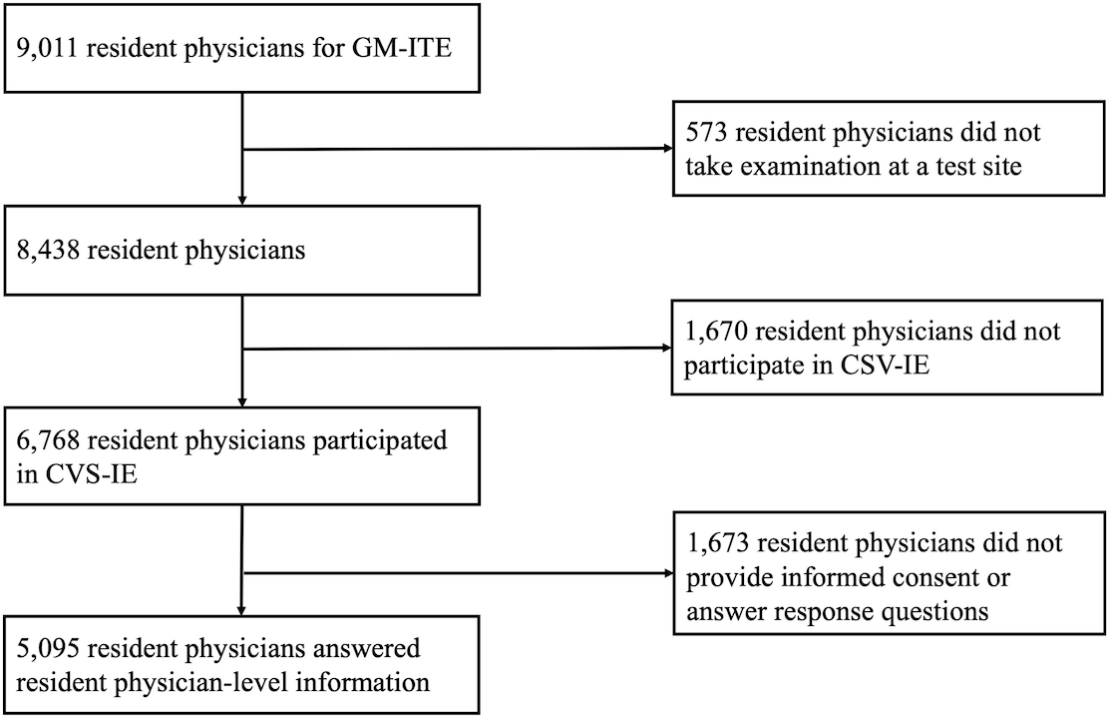
Flowchart of the study. CSV-IE: innovative examination clinical simulation video; GM-ITE: General Medicine In-Training Examination.

Overall, 5105 first- and second-year resident physicians participated in this study (Figure 1). There were no missing data for any covariates or outcome variables. Resident physicians were divided based on their ability to detect tachypnea, the critical non-verbal cue (Table 1). Among the non-verbal cues included in the clinical simulation video, tachypnea exhibited the largest difference in the identification rates between correct and incorrect responders (Multimedia Appendix 2). Overall, tachypnea was overlooked and detected in 4146 and 959 residents, respectively. Before matching, several covariates demonstrated notable imbalances between the tachypnea non-detection and detection groups. Specifically, the proportion of male residents was higher in the non-detection group (69.2% vs 64.7%; SMD=0.219), while the proportion of first-year postgraduate residents was higher in the non-detection group (51.7% vs 45.3%; SMD=0.309). Age distribution also showed discrepancies, with younger residents, particularly those aged 26 years, being more common in the detection group (34.1% vs 31.1%; SMD=0.312). These imbalances were addressed through propensity score matching identifying 959 participants to achieve well-balanced covariates across groups (SMD <0.1 for all covariates).

**Table 1.** Baseline characteristics of the participants included before and after propensity score matching.

Characteristic	All, n (%) (N=5105)	Before propensity score matching			After propensity score matching		
		Tachypnea non-detection group, n (%), (n=4146)	Tachypnea detection group, n (%), (n=959)	SMD	Tachypnea non-detection group, n (%), (n=959)	Tachypnea detection group, n (%), (n=959)	SMD
Sex				0.219			0.015
Men	3488 (68.3)	2867 (69.2)	621 (64.7)		614 (64.0)	621 (64.7)	
Women	1617 (31.7)	1279 (30.8)	338 (35.3)		345 (36.0)	338 (35.3)	
PGY^a^				0.309			0.036
1	2576 (50.5)	2142 (51.7)	434 (45.3)		451 (47.0)	434 (45.3)	
2	2529 (49.5)	2004 (48.3)	525 (54.7)		508 (53.0)	525 (54.7)	
Age (years)				0.312			0.079
24	166 (3.3)	138 (3.3)	28 (2.9)		31 (3.2)	28 (2.9)	
25	1024 (20.1)	816 (19.7)	208 (21.7)		205 (21.4)	208 (21.7)	
26	1618 (31.7)	1291 (31.1)	327 (34.1)		343 (35.8)	327 (34.1)	
27	1060 (20.8)	860 (20.8)	200 (20.9)		208 (21.7)	200 (20.9)	
28	497 (9.7)	419 (10.1)	78 (8.1)		62 (6.8)	78 (8.1)	
29	211 (4.1)	183 (4.4)	28 (2.9)		24 (2.5)	28 (2.9)	
≥30	482 (9.4)	401 (9.7)	81 (8.5)		78 (8.1)	81 (8.5)	
Unknown	47 (0.9)	38 (0.9)	9 (0.9)		8 (0.8)	9 (0.9)	
General medicine rotation				0.118			0.017
No	2341 (45.9)	1860 (44.9)	481 (50.2)		473 (49.3)	481 (50.2)	
Yes	2764 (54.1)	2286 (55.1)	478 (49.8)		486 (50.8)	478 (49.8)	
Internal Medicine rotation (months)				0.237			0.058
0‐5	1362 (26.7)	1117 (26.9)	245 (25.5)		259 (27.0)	245 (25.5)	
6‐10	3149 (61.7)	2552 (61.5)	597 (62.3)		597 (62.3)	597 (62.3)	
11‐15	523 (10.2)	418 (10.1)	105 (11.0)		94 (9.8)	105 (11.0)	
16‐20	53 (1.0)	44 (1.1)	9 (0.9)		6 (0.6)	9 (0.9)	
≥21	18 (0.4)	15 (0.4)	3 (0.3)		3 (0.3)	3 (0.3)	
ED^b^ duty per month				0.103			0.089
0	131 (2.6)	109 (2.6)	22 (2.3)		19 (2.0)	22 (2.3)	
1‐2	841 (16.5)	703 (17.0)	138 (14.4)		129 (13.5)	138 (14.4)	
3‐5	3635 (71.2)	2953 (71.2)	682 (71.1)		710 (74.0)	682 (71.1)	
≥6	480 (9.4)	366 (8.8)	114 (11.9)		95 (9.9)	114 (11.9)	
Unknown	18 (0.3)	15 (0.4)	3 (0.3)		6 (0.6)	3 (0.3)	
Average number of assigned inpatients				0.145			0.063
0‐4	1956 (38.3)	1578 (38.0)	378 (39.4)		386 (40.3)	378 (39.4)	
5‐9	2540 (49.8)	2077 (50.1)	463 (48.3)		466 (48.6)	463 (48.3)	
10‐14	385 (7.5)	306 (7.4)	79 (8.2)		79 (8.2)	79 (8.2)	
≥15	113 (2.2)	87 (2.1)	26 (2.7)		19 (2.0)	26 (2.7)	
Unknown	111 (2.2)	98 (2.4)	13 (1.4)		9 (0.9)	13 (1.4)	
Self-study time per day (minutes)				0.198			0.026
0	2312 (45.3)	1882 (45.4)	430 (44.8)		435 (45.4)	430 (44.8)	
1‐30	1965 (38.5)	1574 (38.0)	391 (40.8)		382 (39.8)	391 (40.8)	
31‐60	580 (11.4)	486 (11.7)	94 (9.8)		94 (9.8)	94 (9.8)	
61‐90	156 (3.0)	126 (3.0)	30 (3.1)		32 (3.3)	30 (3.1)	
≥91	92 (1.8)	78 (1.9)	14 (1.5)		16 (1.7)	14 (1.5)	
Duty (hours per week)				0.089			0.074
Category 1 (<60)	2565 (50.2)	2101 (50.7)	464 (48.4)		464 (48.4)	464 (48.4)	
Category 2 (60–79)	1817 (35.6)	1456 (35.1)	361 (37.6)		361 (37.6)	361 (37.6)	
Category 3 (≥80)	723 (14.2)	589 (14.2)	134 (14.0)		134 (14.0)	134 (14.0)	

a PGY: postgraduate year.

b ED: emergency department.

### GM-ITE Scores

Before matching (Table 2), resident physicians in the tachypnea-detection group exhibited significantly higher GM-ITE scores (mean [SD] 47.6 [8.4] points vs 45.3 [8.0] points), with a mean difference of 2.3 points (95% CI 1.7‐2.8). After propensity score matching, the tachypnea-detection group maintained a higher GM-ITE score (mean [SD] 47.6 [8.4] points vs 45.7 [8.1] points; mean difference 1.9 points [95% CI 1.1‐2.6]). These results were consistent across all subgroup analyses.

**Table 2.** Comparison of General Medicine In-Training Examination (GM-ITE) scores between the tachypnea non-detection and detection groups after propensity score matching.

Characteristic	GM-ITE score, mean (SD)		
	Tachypnea non-detection group	Tachypnea detection group	Difference in the mean value (95% CI)
Before matching			
Total participants	45.3 (8.0)	47.6 (8.4)	2.3 (1.7 to 2.8)
After matching			
Total participants	45.7 (8.1)	47.6 (8.4)	1.9 (1.1 to 2.6)
Subgroup analyses after matching			
Sex			
Male	46.0 (8.4)	48.1 (8.8)	2.1 (1.2 to 3.1)
Female	45.2 (7.6)	46.6 (7.5)	1.4 (0.2 to 2.5)
Grade			
PGY-1^a^	45.2 (7.8)	46.5 (7.9)	1.9 (1.1 to 2.6)
PGY-2	46.2 (8.4)	48.5 (8.6)	2.3 (1.3 to 3.4)
Age (years)			
<27	47.2 (7.8)	48.6 (8.1)	2.6 (1.4 to 3.8)
≥27	43.5 (8.2)	46.2 (8.5)	1.0 (-2.5 to 4.5)
Unknown	42.1 (5.8)	47.8 (8.7)	5.7 (-2.0 to 13.3)

a PGY: postgraduate year.

### Clinical Simulation Video Results

The proportion of correct answers within the tachypnea-detection group was significantly high both prior to matching (39.2% vs 3.1%; difference 36.1% [95% CI 33.0‐39.2]) and following matching (39.2% vs 3.0%; differences 36.2% [95% CI: 32.8‐39.4]; Multimedia Appendix 3). The proportion of correct clinical simulation video answers was consistently greater in the tachypnea detection group across subgroup analyses. Formal interaction tests were conducted to evaluate the association between tachypnea and the correct answer rate divided by sex, PGY level, and age (Multimedia Appendix 4). The interaction tests revealed no statistically significant difference between male and female participants (*P*=.692), indicating that sex did not substantially influence the relationship between the tachypnea detection performance and the correct answer rate. In contrast, a significant interaction was identified between PGY-1 and PGY-2 participants (*P*=.003). Furthermore, a significant interaction was observed between participants aged <27 years and those aged ≥27 years (*P*=.021).

## Discussion

### Principal Findings and Comparison With Previous Works

This study highlights the importance of recognizing non-verbal cues, such as tachypnea, in clinical reasoning. Resident physicians who identified tachypnea achieved significantly higher GM-ITE and correct clinical simulation video answers. Although identifying tachypnea as a non-verbal cue in the clinical simulation video was important, it was not the sole determinant of correct diagnosis. Achieving a correct response required integrating multiple findings, including jugular venous distension, cardiac murmur, and limb injury. Therefore, tachypnea detection was associated with—but not equivalent to—correctly answering the clinical simulation video question. Furthermore, the primary analysis examined the relationship between tachypnea detection and overall GM-ITE scores, which are independent of the video-based assessment and reflect broader clinical knowledge. Consistent results in propensity score-matched and stratified subgroup analyses emphasized the direct relationship between the ability to detect non-verbal information and clinical competence, underlining the need to train resident physicians to observe and interpret non-verbal cues during patient interactions. These skills are essential for accurate diagnosis and effective patient care [14-17].

The substantial GM-ITE score difference between the two groups demonstrates the value of non-verbal cue recognition in clinical competency. Video-based simulations provide a unique opportunity to address this gap, offering realistic scenarios that mirror actual clinical encounters [18]. By integrating non-verbal with verbal information, video simulations enhance diagnostic capabilities potentially underdeveloped through text-based evaluations alone.

Our findings further indicated that clinical experience, as reflected in PGY, significantly influence the ability to recognize non-verbal cues. PGY-2 residents outperformed PGY-1 residents, indicating that experiential learning plays a pivotal role in developing these perceptual skills [19]. Beyond the PGY level, analysis of the unmatched cohort revealed additional factors associated with improved cue recognition. For example, the detection group more frequently reported a strong interest in general medicine and prior experience with video-based clinical reasoning, indicating that both motivational and educational factors may enhance observational accuracy. Furthermore, residents with greater monthly patient exposure demonstrated higher detection rates, indicating that regular clinical exposure helps refine perceptual skills. Together, these findings highlight several modifiable factors that could be targeted through future educational interventions. Further research should explore how medical training and curricula can be optimized to foster the development of diagnostic observation skills early in clinical education.

Including video-based assessments in the GM-ITE reinforces its potential to complement traditional MCQs. Video simulations enable a more comprehensive evaluation of diagnostic abilities by integrating complex, real-world scenarios [20]. They assess clinical knowledge and challenge resident physicians to process subtle cues, bridging the gap between theoretical understanding and practical application [21-23]. This also reinforces the value of integrating video-based assessments with traditional MCQ examinations to comprehensively evaluate a physician’s diagnostic capabilities [21,22]. Overall, this finding suggests potential areas for further investigation into whether perceptual abilities, training experiences, or other underlying factors contribute to differences [24]. The significant proportion of second-year resident physicians in the detection group indicates the impact of clinical experience on non-verbal cue recognition, emphasizing the importance of developing skills during medical training.

Although our results showed that resident physicians who detected tachypnea also had higher GM-ITE scores; this association does not indicate redundancy between video- and text-based assessments. Rather, it suggests that the ability to recognize non-verbal cues is an important component of clinical reasoning that complements traditional knowledge-based assessments. GM-ITE MCQs primarily evaluate declarative knowledge and structured reasoning, whereas the clinical simulation video captures real-time perceptual and interpretive skills, such as visual observation and contextual integration. Therefore, this video-based format offers added value by assessing diagnostic competencies that may not be fully captured through text-based questions alone.

Using video-based simulations like clinical simulation videos significantly enhances traditional MCQ examinations by enabling the assessment of resident physicians’ ability to integrate verbal and non-verbal information, thereby replicating real-world clinical scenarios, and improving the assessment of clinical competence [24]. Our findings support the inclusion of video-based simulations in medical education assessments [21]. Video simulations can better prepare resident physicians for the complexities of patient care by mimicking actual clinical situations [25,26]. However, future studies should investigate how repeated exposure to video-based assessments impacts diagnostic accuracy and confidence, as well as their influence on clinical outcomes.

The consistency in GM-ITE scores and clinical simulation video results after propensity score matching and subgroup stratification indicated the robustness of our findings [27]. Furthermore, our findings advocate for the broader adoption of video simulations in medical education, as they offer an innovative and effective means to improve diagnostic skills and preparedness among future clinicians [20,28].

In addition to repeated exposure to video-based examinations, future research should explore methods of training human raters to reliably assess non-verbal cue detection. Standardized rater-training programs or calibration protocols should ensure consistent evaluations in video-based objective structured clinical examination-type assessments. Furthermore, advances in artificial intelligence and machine learning could enable the automated recognition of visual and auditory cues in clinical simulations, supporting scalable and objective assessment of non-verbal diagnostic skills.

### Limitations

This study has certain limitations. First, the cross-sectional design limited our ability to infer causality between the detection of non-verbal cues like tachypnea and higher GM-ITE scores. Future research should consider longitudinal or interventional study designs, to more definitively evaluate causality. Specifically, intervention studies could assess whether targeted training in non-verbal cue recognition—such as simulation-based educational programs—facilitates measurable improvements in the diagnostic accuracy and GM-ITE performance. Longitudinal approaches may clarify whether enhanced cue recognition skills contribute to sustained improvements in clinical competence. Second, the study relied on self-reported data from resident physicians, which may be subject to response bias. Although we used objective measures such as the GM-ITE scores, the accuracy of the self-reported demographic and training information could not be independently verified. Third, this study was conducted in Japan, where medical education and training systems may differ from other countries. Consequently, these findings may not be generalizable to resident physicians in different healthcare systems with varying educational structures. However, the highlighted diagnostic challenges—particularly the recognition or interpretation of subtle clinical cues and prevention of diagnostic errors—are likely to be relevant across a broad range of training contexts [29]. Therefore, while the GM-ITE context is unique, our findings offer insights that may resonate with global generalist training programs. Fourth, although propensity score matching was applied to control potential confounders, unmeasured variables may have influenced the results. While propensity score matching strengthened the validity of our findings by controlling for potential confounders [30], unmeasured factors, such as prior training in video simulation or baseline observational skills, may still influence the results [31]. A deeper understanding of these variables could provide further insights into the mechanisms underlying the differences in GM-ITE scores. Finally, the innovative examination using video simulations was a relatively new GM-ITE addition, and the novelty of the format might have influenced participants’ performance. Ongoing evaluation of the reliability and validity of video-based assessments is essential to ensure their effectiveness as tools for measuring clinical competence.

### Conclusions

The ability to recognize non-verbal cues, such as tachypnea, is a critical determinant of clinical competence among resident physicians, as evidenced by higher GM-ITE scores. Video-based simulations exhibit a transformative addition to traditional MCQ examinations, enabling a comprehensive evaluation of diagnostic abilities by integrating real-world complexities and non-verbal information.

## Supplementary material

10.2196/72640 Multimedia Appendix 1 Sample multiple-choice question from the General Medicine In-Training Examination (GM-ITE).

10.2196/72640 Multimedia Appendix 2 Detection rates of non-verbal clinical findings in the simulation video based on answer correctness.

10.2196/72640 Multimedia Appendix 3 Comparison of the correct clinical simulation video answers between the tachypnea non-detection and detection groups after propensity score matching.

10.2196/72640 Multimedia Appendix 4 Relationship between tachypnea detection and correct clinical simulation video answer, interaction tests.

## Abbreviations

GM-ITE: general medicine in-training examination
IM-ITE: internal medicine residency examination
JAMEP: Japan Institute for Advancement of Medical Education Program
MCQs: multiple-choice questions
PGY: postgraduate year
