# Supplementary material for: Resident Physician Recognition of Tachypnea in Clinical Simulation Videos in Japan: Cross-Sectional Study
Source: JMIR Med Educ. 2025 Jul 31;11:e72640. doi: 10.2196/72640 (PMC12313080; doi:10.2196/72640)
Supplement: Multimedia Appendix 1 [file mededu-v11-e72640-s001.docx]

**Question**

An 18-year-old man is brought to the hospital following a head-on collision with a car while riding his bicycle 15 minutes prior. The car was traveling at about 40 kph. When the paramedics arrived at the site, the patient was lying supine on the ground approximately 5 meters from his bicycle. His GCS and vital signs, as measured by the paramedics, are as follows: GCS is E3V4M6, blood pressure is 90/48 mmHg, heart rate is 120 beats per minute, respiratory rate is 20 breaths per minute, and SpO_2_ is 100% on 10 L/minute via O_2_ mask with a reservoir bag. His GCS and vital signs on arrival to the emergency department are similar to those determined by the paramedics. His airway is open. He is not in respiratory distress, there are no abnormal breathing sounds, and thoracic motion is symmetrical. There is no subcutaneous emphysema. Abdominal findings are normal. His scrotum is enlarged and has purpura. There is no apparent limb deformity. He has peripheral coldness. FAST assessment is negative.

Which of the following most likely explains the patient’s shock status?

(1)　Spinal cord injury

(2)　Tension pneumothorax

(3)　Retroperitoneal hemorrhage

(4)　Severe head trauma

(5)　Cardiac tamponade

**Answer**

(3)　Retroperitoneal hemorrhage
