# Supplementary material for: Resident Physician Recognition of Tachypnea in Clinical Simulation Videos in Japan: Cross-Sectional Study
Source: JMIR Med Educ. 2025 Jul 31;11:e72640. doi: 10.2196/72640 (PMC12313080; doi:10.2196/72640)
Supplement: Multimedia Appendix 2 [file mededu-v11-e72640-s002.docx]

| Clinical finding | Correct clinical simulation video answer group (n = 504) | Incorrect clinical simulation video answer group (n = 4601) |
| --- | --- | --- |
| Tachypnea | 375 (74.4%) | 440 (9.6%) |
| Jugular venous distension | 375 (74.4%) | 1058 (23.0%) |
| Right lower limb fracture | 133 (26.4%) | 64 (1.4%) |
| Cardiac murmur | 119 (23.6%) | 2126 (46.2%) |
