# Supplementary material for: Resident Physician Recognition of Tachypnea in Clinical Simulation Videos in Japan: Cross-Sectional Study
Source: JMIR Med Educ. 2025 Jul 31;11:e72640. doi: 10.2196/72640 (PMC12313080; doi:10.2196/72640)
Supplement: Multimedia Appendix 3 [file mededu-v11-e72640-s003.docx]

|  | Correct clinical simulation video answer, n (%) | |  | |
| --- | --- | --- | --- | --- |
|  | Tachypnea non-detection  group | Tachypnea detection  group | Differences in proportion,  % (95%CI) | |
| Before matching | n = 4146 | n = 959 |  | |
| Total participants | 129 (3.1) | 376 (39.2) | 36.1 (33.0, 39.2) | |
| After matching | n = 959 | n = 959 |  | |
| Total participants | 29 (3.0) | 376 (39.2) | 36.2 (32.8, 39.4) | |
| Subgroup analyses after matching | |  |  |  |
| **Gender** |  |  |  |  |
| Male | 21 (3.4) | 255 (41.1) | 37.6 (33.4, 41.7) |  |
| Female | 8 (2.3) | 121 (35.8) | 33.5 (27.9, 38.7) |  |
| **Grade** |  |  |  |  |
| PGY-1 | 18 (4.0) | 141 (32.5) | 28.5 (23.6, 33.1) |  |
| PGY-2 | 11 (2.2) | 235 (44.7) | 42.6 (38.0, 46.9) |  |
| **Age (year)** |  |  |  |  |
| <27 years | 25 (4.3) | 228 (40.5) | 36.2 (31.7, 40.4) |  |
| ≥27 years | 4 (1.1) | 145 (37.5) | 36.4 (31.2, 41.2) |  |
| Unknown | 0 (0) | 3 (33.3) | 33.3 (-7.6, 60.3) |  |

CI, confidence interval; PGY, postgraduate year
