# Supplementary material for: Resident Physician Recognition of Tachypnea in Clinical Simulation Videos in Japan: Cross-Sectional Study
Source: JMIR Med Educ. 2025 Jul 31;11:e72640. doi: 10.2196/72640 (PMC12313080; doi:10.2196/72640)
Supplement: Multimedia Appendix 4 [file mededu-v11-e72640-s004.docx]

| **Subgroup** | **Tachypnea detection** | **Correct clinical simulation video answer** | **n** | **Prevalence** | **Odds ratio (95%CI)** | **p-value for interaction** |
| --- | --- | --- | --- | --- | --- | --- |
| Gender |  |  |  |  |  |  |
| Male | Detection | 255 | 621 | 41 % | 19.67 (12.37 – 31.28) | 0.692 |
|  | Non-detection | 21 | 614 | 3 % |  |  |
| Female | Detection | 121 | 338 | 36 % | 23.49 (11.26 – 49.01) |  |
|  | Non-detection | 8 | 345 | 2 % |  |  |
| Grade |  |  |  |  |  |  |
| PGY-1 | Detection | 141 | 434 | 32 % | 11.58 (6.93 – 19.36) | 0.003 |
|  | Non-detection | 18 | 451 | 4 % |  |  |
| PGY-2 | Detection | 235 | 525 | 45 % | 36.61 (19.66 – 68.18) |  |
|  | Non-detection | 11 | 508 | 2 % |  |  |
| Age (year) |  |  |  |  |  |  |
| <27 years | Detection | 228 | 563 | 40 % | 15.08 (9.77 – 23.29) | 0.021 |
|  | Non-detection | 25 | 579 | 4 % |  |  |
| ≥27 years | Detection | 145 | 387 | 37 % | 55.12 (20.15 – 150.82) |  |
|  | Non-detection | 4 | 372 | 1 % |  |  |

CI, confidence interval; PGY, postgraduate year
